# Mutations in Gamma Adducin are Associated With Inherited Cerebral Palsy

**DOI:** 10.1002/ana.23971

**Published:** 2014-01-21

**Authors:** Michael C Kruer, Tyler Jepperson, Sudeshna Dutta, Robert D Steiner, Ellen Cottenie, Lynn Sanford, Mark Merkens, Barry S Russman, Peter A Blasco, Guang Fan, Jeffrey Pollock, Sarah Green, Randall L Woltjer, Catherine Mooney, Doris Kretzschmar, Coro Paisán-Ruiz, Henry Houlden

**Affiliations:** 1 Sanford Children's Health Research CenterSioux Falls, SD; 2 Department of Molecular and Medical Genetics, Oregon Health and Science UniversityPortland, OR; 3 Department of Pediatrics, Oregon Health and Science UniversityPortland, OR; 4 Department of Molecular Neuroscience, University College London Institute of NeurologyLondon, United Kingdom; 5 Child Development and Rehabilitation Center, Oregon Health and Science UniversityPortland, OR; 6 Department of Neurology, Portland Shriner's HospitalPortland, OR; 7 Division of Hematopathology, Department of Pathology, Oregon Health and Science UniversityPortland, OR; 8 Division of Neuroradiology, Department of Radiology, Oregon Health and Science UniversityPortland, OR; 9 Division of Neuropathology, Department of Pathology, Oregon Health and Science UniversityPortland, OR; 10 Complex and Adaptive Systems Laboratory, School of Medicine and Medical Science, University College DublinDublin, Ireland; 11 Departments of Neurology, Psychiatry, and Genetics and Genomic Sciences, Icahn School of Medicine at Mount SinaiNew York, NY; 12 Friedman Brain and Mindich Child Health and Development Institutes, Icahn School of Medicine at Mount SinaiNew York, NY

## Abstract

**Objective:**

Cerebral palsy is estimated to affect nearly 1 in 500 children, and although prenatal and perinatal contributors have been well characterized, at least 20% of cases are believed to be inherited. Previous studies have identified mutations in the actin-capping protein KANK1 and the adaptor protein-4 complex in forms of inherited cerebral palsy, suggesting a role for components of the dynamic cytoskeleton in the genesis of the disease.

**Methods:**

We studied a multiplex consanguineous Jordanian family by homozygosity mapping and exome sequencing, then used patient-derived fibroblasts to examine functional consequences of the mutation we identified in vitro. We subsequently studied the effects of adducin loss of function in *Drosophila*.

**Results:**

We identified a homozygous c.1100G>A (p.G367D) mutation in *ADD3*, encoding gamma adducin in all affected members of the index family. Follow-up experiments in patient fibroblasts found that the p.G367D mutation, which occurs within the putative oligomerization critical region, impairs the ability of gamma adducin to associate with the alpha subunit. This mutation impairs the normal actin-capping function of adducin, leading to both abnormal proliferation and migration in cultured patient fibroblasts. Loss of function studies of the *Drosophila* adducin ortholog *hts* confirmed a critical role for adducin in locomotion.

**Interpretation:**

Although likely a rare cause of cerebral palsy, our findings indicate a critical role for adducins in regulating the activity of the actin cytoskeleton, suggesting that impaired adducin function may lead to neuromotor impairment and further implicating abnormalities of the dynamic cytoskeleton as a pathogenic mechanism contributing to cerebral palsy.

Cerebral palsy (CP) is a relatively common cause of neuromotor disability, estimated to affect approximately 1 in 500 live births.^1^ Many cases of CP are thought to result from prenatal or perinatal insults, identifiable on magnetic resonance imaging (MRI) scans by periventricular leukomalacia, diffuse white matter injury, or encephalomalacia. However, approximately 20% of children with CP have no identifiable etiology for their symptoms, with normal or nonspecific findings on neuroimaging, suggesting that a significant number of cases may be heritable in nature.^2^ Thus far, several genes responsible for inherited forms of spastic quadriplegic CP have been identified, including the actin-capping protein *KANK1* (Mendelian Inheritance in Man [MIM] # 612900)^3^ and the adaptor protein complex-4 subunits *AP4E1*, *AP4M1*, *AP4B1*, and *AP4S1* (MIM # 612936, 613744, 614066, 614067),^4^–^6^ responsible for both endocytic and secretory sorting of integral membrane proteins.

In mammalian systems, adducin proteins form complexes with actin-spectrin, linking membranes to the dynamic cytoskeleton. Adducins have a physiologic actin-capping function, modulating the fast-growing end of actin filaments and controlling actin molecule length. Prior studies have shown adducins to have effects on plasma membrane stability,^7^ cell motility,^8^ synaptic vesicle/endosomal recycling,^9^ cell–cell adhesion,^10^ cell proliferation,^11^ and dendritic spine formation and retraction.^12^ An α adducin polymorphism (rs4961) has been linked to CP in prior association studies,^13^ and adducin hyperphosphorylation has been identified in both human patients and mouse models of amyotrophic lateral sclerosis.^14^ Here, we describe the characterization of a defect in the adducin subunit gene *ADD3* in a consanguineous family with spastic diplegic or quadriplegic CP.

## Patients and Methods

### Ethics Statement

Written informed consent was provided for all participants using an institutional review board–approved protocol.

### Index Family

**FIGURE 1:**
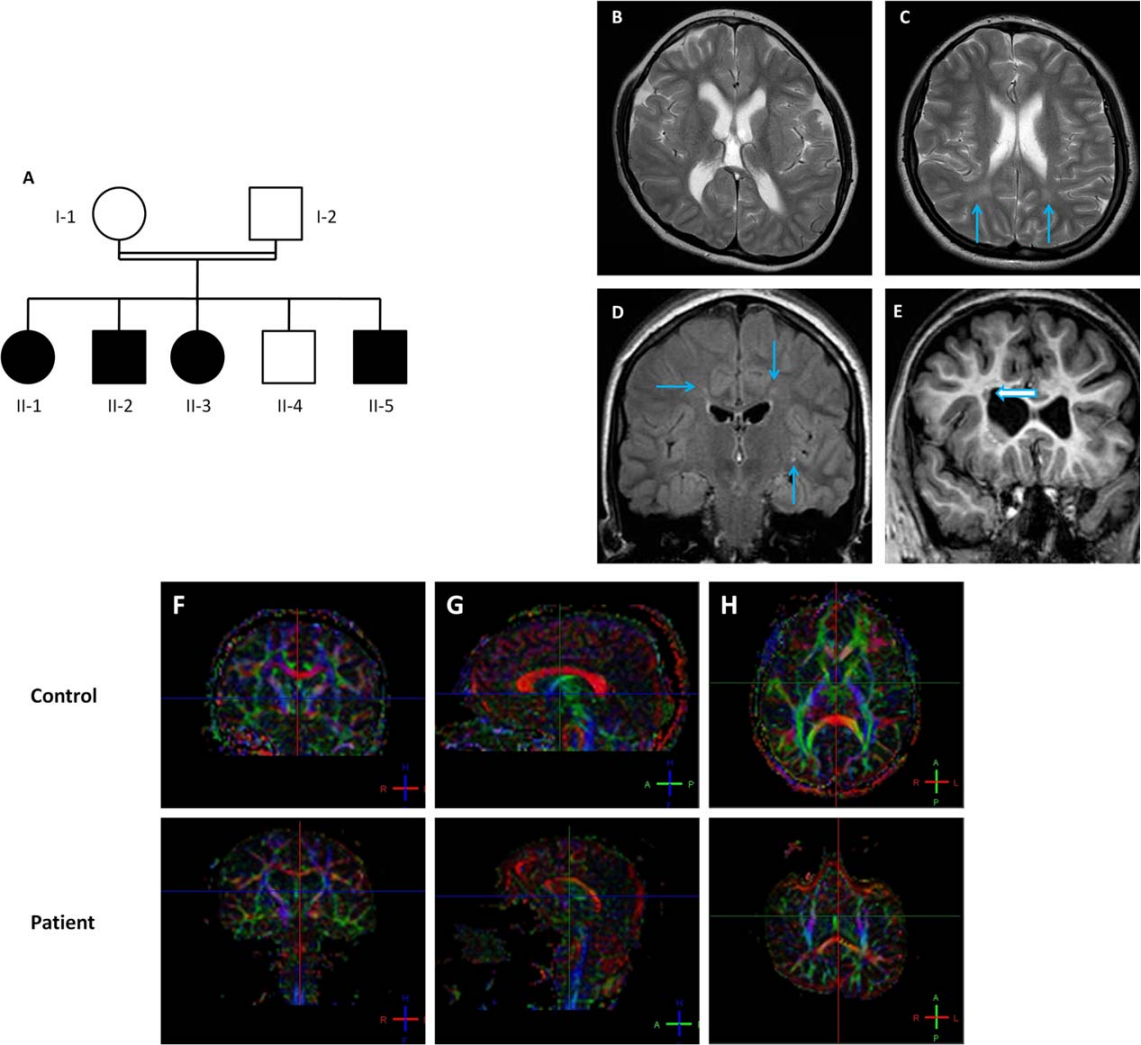
Pedigree and neuroimaging findings of index family. (A) Family structure. (B) Frontotemporal predominant volume loss was observed, with decreased white matter volume and mild ventriculomegaly in all family members. (C, D) Tiny periventricular T2 hyperintense foci were seen *(arrows)* in all family members. (E) Periventricular heterotopic gray matter was noted in patient II-2 *(arrow)*. (F–H) Sagittal fractional anisotropy (FA) map generated from diffusion tensor imaging through the corpus callosum in control (above) and Patient II-2 (below). Color maps are based on major eigenvector orientation in each of the voxels: red = right (R) to left (L), green = anterior (A) to posterior (P), blue = superior–inferior anatomical directions. When compared to an age-/sex-matched control, substantial differences in the FA values of the body (0.413 ± 0.194 [patient] vs. 0.799 ± 0.087 [control]) of the corpus callosum were evident. [Color figure can be viewed in the online issue, which is available at http://www.annalsofneurology.org.]

The index family was a consanguineous (second-cousin marriage) Jordanian family with 4 affected individuals (Fig 1A). Parents were examined and were unaffected. All children were born at term after unremarkable pregnancies.

### Patient Phenotype

No facial dysmorphism was evident. Spasticity was noted in Patient II-1 at 3 months of age. Borderline microcephaly was present (occipitofrontal circumference < 3%). Communicative intent was limited to nodding yes/no and occasional vocalization. Intermittent icterus developed during intercurrent illness associated with mild conjugated hyperbilirubinemia. Nerve conduction study and electromyogram were normal. At age 16 years, she was nonambulatory, with severe spastic quadriplegia and pyramidal tract signs without dystonia. She was gastrostomy tube-fed and dependent for all activities of daily living.

The second sibling (II-2) also exhibited spastic quadriparesis and cognitive impairment within the first year. He developed epilepsy by age 2 years, which responded to anticonvulsant medications. Electroencephalogram demonstrated bifrontal spike–slow wave discharges. Supranuclear gaze palsy was noted during the preschool years but improved with time. Intermittent conjugated hyperbilirubinemia developed during illness. Examination at age 13 years showed borderline microcephaly, dysarthria, dysphagia, and exotropia. Electron microscopic analysis of a skin biopsy showed normal morphology without inclusions. Bone marrow biopsy performed at age 8 years showed sideroblastic anemia, although peripheral smear with electron microscopy at age 14 years found no anemia or ringed sideroblasts.

The third child (II-3) was more mildly affected, with spastic diplegia (Supplementary Video). Neuropsychological evaluation disclosed borderline intellectual functioning, inattention, and impulsivity. At age 7 years, examination found borderline microcephaly, strabismus, and spastic diplegia. A single episode of conjugated hyperbilirubinemia occurred after orthopedic surgery.

The fourth child (II-4) was phenotypically normal at age 5 years.

The fifth child (II-5) showed early cognitive and neuromotor impairment. His examination at age 22 months demonstrated convergence retraction nystagmus and strabismus. He had borderline microcephaly, dysphagia, and spastic quadriparesis with pyramidal tract signs. Receptive understanding was severely limited, and expressive speech was absent.

### Diagnostic Workup

Diagnostic testing included normal karyotype and array comparative genomic hybridization. Complete blood count showed intermittent macrocytic, normochromic anemia in several affected children. Metabolic screening was unremarkable.

Muscle biopsy performed in II-1 and II-2 demonstrated mild type 2 fiber atrophy with unremarkable histologic and electron microscopic appearance. Respiratory chain enzymology in frozen muscle demonstrated a mild decrease in complex I activity (mU/U citrate synthase (CS)). Analysis for mtDNA deletions, duplications, and depletion and screens for MELAS (mitochondrial encephalomyopathy, lactic acidosis, and strokelike episodes), MERRF (myoclonic epilepsy with ragged red fibers), and NARP (neuropathy, ataxia, and retinitis pigmentosa) were negative.

Phenotypic features and workup are summarized in Table 1. MRI features are depicted in Figure 1B–H and Table 2. Spinal cord imaging was unremarkable.

**TABLE 1:** Clinical Features of Affected Family Members

Feature	II-1	II-2	II-3	II-5
Sex	F	M	F	M

Age at last examination, yr	16	13	9	3

Neuromotor findings	Spastic quadriplegia	Spastic quadriplegia	Spastic diplegia	Spastic quadriplegia

Intellectual disability	Severe	Severe	Borderline	Moderate

MRI	Frontoparietal volume loss; punctate T2 hyperintensities	Periventricular T2 hyperintensities; GM heterotopia	Normal	Normal

EEG	ND	Bifrontal spike-wave	ND	ND

EMG/NCS	Normal	ND	ND	ND

Other findings		Supranuclear gaze palsy	ADHD	Convergence-retraction nystagmus

ADHD = attention deficit–hyperactivity disorder; EEG = electroencephalogram; EMG = electromyogram; F = female; GM = gray matter; M = male; MRI = magnetic resonance imaging; NCS = nerve conduction study; ND = not done.

**TABLE 2:** Fractional Anisotropy Values of Major White Matter Tracts

	Control	II-2
ALIC	0.506 ± 0.142	0.515 ± 0.176

BODY	0.582 ± 0.112	0.413 ± 0.194

EC	0.341 ± 0.143	0.324 ± 0.111

GENU	0.799 ± 0.087	0.455 ± 0.203

PLIC	0.619 ± 0.166	0.679 ± 0.163

ALIC = anterior limb of internal capsule; EC = external capsule; PLIC = posterior limb of internal capsule.

### Cell Culture

Fibroblast cell lines were established from patient skin biopsies. Low-passage, age-matched cells were used for all experiments.

### Neuroimaging

MRI images were obtained using a 3T Philips (Best, the Netherlands) Achieva Scanner, with diffusion tensor imaging postprocessing via the Fibertrack Package (Philips).

### Genotyping

Genotyping was performed using HumanCytoSNP-12 BeadChips (Illumina, San Diego, CA), and homozygous regions were annotated as previously described.^15^

### Exome Sequencing

Coding sequence was captured using the SureSelect exome capture kit (Agilent, Santa Clara, CA). Adapter sequences were ligated, and enriched DNA samples were analyzed on the GAIIx (Illumina). Paired-end reads of 42bp lengths were produced. Sequence alignment; building, indexing, and sorting SAM (Sequence Alignment/Map) files; and calling raw variants and indels were performed using Burrows–Wheeler Aligner (http:// io-bwa.sourceforge.net/), SAMTools (http://samtools.sourceforge.net/), and the Genomic Analysis Toolkit (https://www.broadinstitute.org/gsa/wiki/index.php/TheGenomeAnalysisToolkit) and Picard (http://picard.sourceforge.net/), respectively. Approximately 73.5 million sequence reads were produced per sample, with on-target coverage of 98.75% of reads mapped to hg18. Average read-depth was ×56; in the chromosome 10 region of shared homozygosity identified by single nucleotide polymorphism (SNP) genotyping, average coverage depth was ×80. A total of 14,427 nonunique, 12,627 unique, and 1,640 indel sequence variants were detected. To assess variant functional impact, we applied phastCons and PolyPhen-2.^16^,^17^

### Sanger Sequencing

Genomic DNA was polymerase chain reaction–amplified (sequences available upon request) and sequenced on an ABI3730 sequencer (Life Technologies, Carlsbad, CA).

### In Silico Analysis

Short linear protein-binding motifs (SLiMs) were predicted using SLiMPred (http://bioware.ucd.ie/∼compass/biowareweb/).^18^ Protein intrinsic disorder was predicted with IUPred (http://iupred.enzim.hu/).^19^ Three-class protein secondary structure (helix, strand, and coil) was predicted by Distill (http://distill.ucd.ie/).^20^

### Actin Capping

Actin-capping assays were performed using an Actin Polymerization Biochem Kit (Cytoskeleton, Denver, CO).^21^ Pyrene-labeled G-actin was incubated with cell lysates after 1μg/ml lysophosphatidic acid stimulation for 2 hours.^22^

### Colocalization

Fibroblasts were grown to near confluence, and cells were fixed in 4% paraformaldehyde, treated with mouse anti-ADD1 (1:500; Abcam, Boston, MA) and rabbit anti-ADD3 (Novus Biologicals, Littleton, CO), and visualized using Alexa Fluor fluorophores (Life Technologies, Carlsbad, CA). Slides were mounted using Vectashield H-1200 with DAPI (Vector Laboratories, Burlingame, CA), and z-stack images were taken using an A1 TIRF confocal microscope (Nikon, Tokyo, Japan). Colocalization was determined using the Nikon NIS Colocalization module.

### Proliferation and Migration

Cells were grown on poly-L-lysine coverslips (BD Biosciences, Franklin Lake, NJ). A clear channel was mechanically introduced, and the number of cells within the clearing was counted.

Proliferation was estimated by the percentage of Ki-67–positive cells, whereas migration was determined by subtracting proliferating cells from total cell content within the region of interest. For immunocytochemistry, cells were fixed and stained as above, using anti-Ki-67 (1:300; Millipore, Billerica, MA).

### siRNA Knockdown

Control fibroblasts were treated with 75nM siRNA duplexes targeted against human *ADD3* (Origene, Rockville, MD) using RNAiMAX (Invitrogen, Carlsbad, CA) for 48 hours. Transfection efficiency was determined using an fluorescein isothiocyanate–conjugated control (Life Technologies #2013) run in parallel. Cell lysates were used for ADD3 immunoblot and actin polymerization assay.

### Endocytosis

Fibroblasts were transferred to ice-cold F12 with 50μg/ml Alexa Fluor 488-transferrin. Cells were washed to remove excess label, then incubated for 30 minutes at 37°C to allow for transferrin uptake. Cells were fixed with 2% paraformaldehyde, mounted with Prolong Gold Antifade and imaged using a 710 confocal microscope (Carl Zeiss, Oberkochen, Germany). Image processing and evaluation were performed using Volocity (Perkin Elmer, Waltham, MA).

### *Drosophila* Stocks

Strains were Cs (wild type), hts01103, Df(2R) BSC26 (Bloomington Stock Center, Bloomington, IN), UAS-hts RNAi (line 103631; Vienna Drosophila RNAi Center, Vienna, Austria). Applx-Gal4 was kindly provided by L. Torroya.

### Western Blots

Immunoblotting used anti–adducin-related protein (1B1; 1:10) and anti–beta-tubulin (E7; 1:50; Developmental Hybridoma Bank, Iowa City, IA) and peroxidase-conjugated sheep antimouse secondary antibody (1:10,000; Jackson laboratories, Bar Harbor, ME).^23^ Fly head homogenates were prepared from 3- to 6-day-old flies, and hts protein was detected using the ECL system (GE Healthcare, Buckinghamshire, United Kingdom).

### Paraffin Sections

Fly heads were fixed and embedded in paraffin. Seven-micrometer sections were deparaffinized, mounted, and imaged using a Zeiss Axioscope 2 microscope. Autofluorescence due to endogenous eye pigmentation allowed visualization.^24^

### *Drosophila* Behavioral Assay

The negative geotaxis assay was performed by transferring flies to a graduated vial and tapping to the bottom of the vial.^25^ Percentages of flies climbing 8cm within 8 seconds post-tap were recorded.

## Results

### Homozygosity Mapping

**FIGURE 2:**
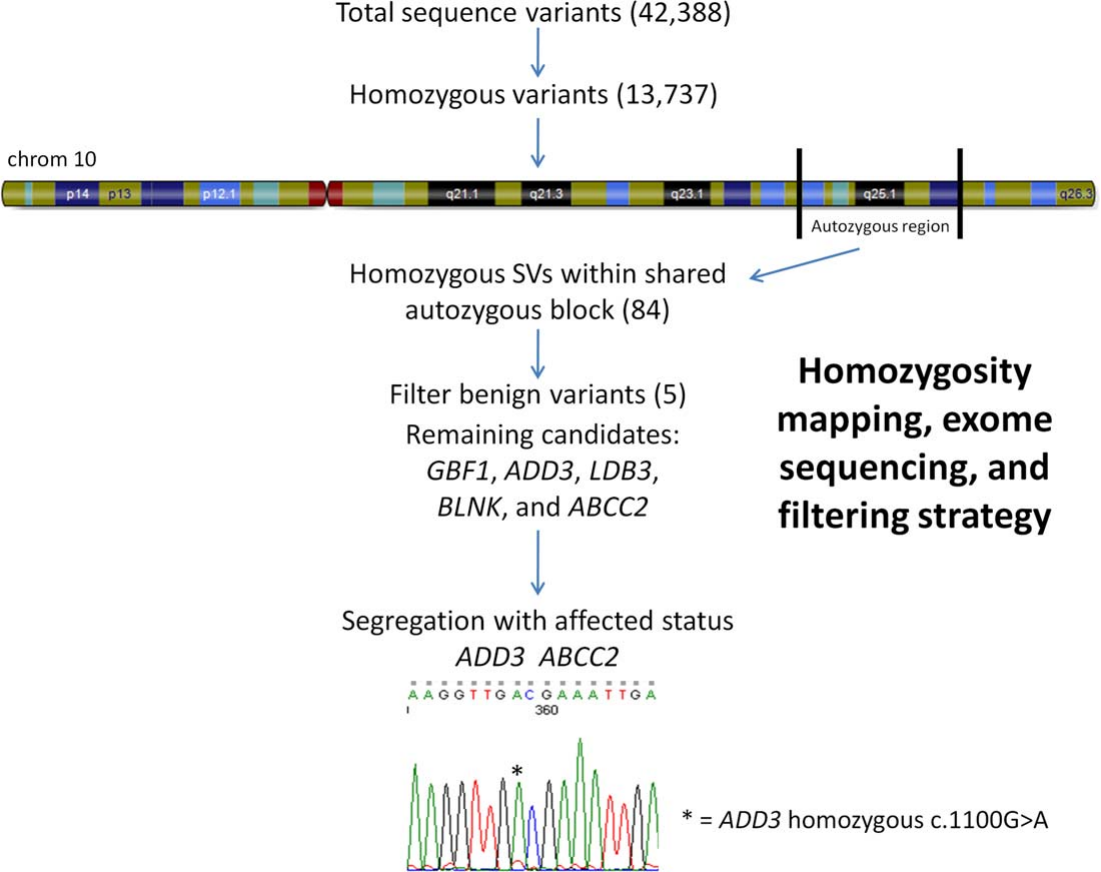
Homozygosity mapping, exome and Sanger sequencing, and filtering algorithm. Homozygous region on chromosome 10 shared by affected family members and filtering strategy applied to interrogate sequence variants (SVs) generated by exome sequencing. A homozygous c.1100G>A mutation was found in all affected individuals, whereas unaffected individuals were heterozygous for this mutation. [Color figure can be viewed in the online issue, which is available at http://www.annalsofneurology.org.]

Given the inheritance pattern of the family, we performed autozygosity mapping using llumina HumanCytoSNP-12 BeadChips in all family members. We identified 134 autozygous segments that were ≥1Mb; however, only 2 autozygous blocks on chromosome 10 were common to all affected individuals and absent in unaffected family members (physical position 99,527,480–103,563,212, and 105,450,391–114,830,484; hg18; Fig 2).

### Exome Sequencing

Given the size of the autozygous region identified, we applied a whole exome sequencing approach to identify the causative variant in this family by sequencing individual II-2. We systematically filtered sequence variants by focusing on homozygous variants within the linkage region and filtering out known SNPs present in public databases and in control exomes.^26^ Within the linked regions of chromosome 10, on-target coverage was 99.24%. Using this approach, we identified homozygous variants in 5 genes (*GBF1*, *ADD3*, *LDB3*, *BLNK*, and *ABCC2*) that fell within the chromosome 10 autozygous regions. Subsequent Sanger sequencing found that homozygous mutations in only 2 of these genes, *ADD3* (c.1100G>A; ENST00000356080) and *ABCC2* (c.4025C>A; ENST00000370449), segregated with disease status in the family (see Fig 2).

Mutations in *ABCC2* (ENSG00000023839) cause Dubin–Johnson syndrome (MIM # 237500), a well-characterized autosomal recessive disorder that leads to episodic jaundice and conjugated hyperbilirubinemia in times of metabolic stress. As *ABCC2* mutations have never been associated with a neurological phenotype, we propose that the novel homozygous mutation (p.S1342Y) we identified in this gene cosegregates with the gene responsible for the neurologic phenotype, residing within the same haplotype block and accounting for the episodic jaundice seen in affected family members.

### Mutation Validation and In Silico Analysis

In contrast, the c.1100G>A (p.G367D) mutation identified in *ADD3* (ENSG00000148700) represented a viable candidate gene for the neurological phenotype in this family, with both parents carrying heterozygous mutations. Analysis of 208 ethnically matched Middle Eastern controls and 450 mixed European controls revealed no variants at this site. The ADD3 p.G367D mutation occurred at a highly conserved residue that maps within the putative oligomerization domain^27^ of the adducin protein (Supplementary Fig 1). In silico analysis using Distill revealed that the p.G367D mutation was predicted to disrupt the naturally occurring helix surrounding the mutation site. This was associated with widespread disruption of SLiMs as predicted by SLiMPred. This occurred both at the mutation site (oligomerization domain) and throughout the disordered region of the tail and MARCKS domains (actin-binding domain).

### Actin-Capping Studies

**FIGURE 3:**
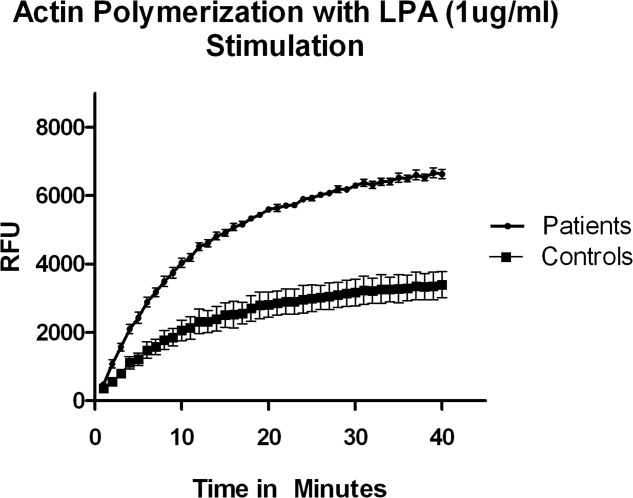
Mutation effects on actin-capping function. Lysophosphatidic acid (LPA)-stimulated actin polymerization is increased in mutant fibroblasts, consistent with impaired adducin-mediated actin capping. Shown are mean values for fibroblast lysates from 3 patients versus 3 age-matched controls run in duplicate, representing results from 2 independent experiments, with mean ± standard deviation depicted; *p* < 0.0001 by Student *t* test. RFU = relative fluorescence units.

Immunoblot analysis of fibroblast lysates from Patients II-1, II-2, and II-3 showed no difference in protein abundance between wild-type and affected individuals (data not shown). Similarly, no significant differences were detected in levels of phosphorylated adducin (data not shown). Immunohistochemistry of affected muscle biopsy samples (II-1 and II-2) as well as confocal microscopic analysis of affected cultured fibroblasts showed no consistent difference in either the expression or distribution of gamma adducin (data not shown). However, fibroblast lysates demonstrated a significant increase in lysophosphatidic acid–stimulated actin polymerization in patient fibroblasts when compared to wild type (Fig 3), consistent with impaired actin capping activity in the mutant. siRNA knockdown of the *ADD3* transcript led to a similar impairment of actin capping (Supplementary Fig 2), suggesting that the p.G367D variant serves as a loss-of-function mutation. Adducin normally functions as a heterodimer or heterotetramer comprised of α/β or α/γ subunits that serve to cap and bundle actin filaments, and recruit spectrin.^27^ Both of these domains are predicted to be disrupted by the p.G367D mutation (see Supplementary Fig 1C), although adducin monomers retain some actin-capping activity within the neck–tail domain.^28^

### Adducin Oligomerization

Recently, α adducin has been shown to traffic between the nucleus and cytoplasm and to play a role in cell proliferation.^11^ Whereas the α and γ subunits are ubiquitously expressed, β subunit expression is limited to brain and hematopoietic tissue. Adducin subunit composition seems to finely regulate adducin activities, having effects on long-term potentiation and motor function as well as the phosphorylation status of individual subunits.^29^ For this reason, we sought to characterize the effects of ADD3 p.G367D on the interaction of the α and γ subunits.

**FIGURE 4:**
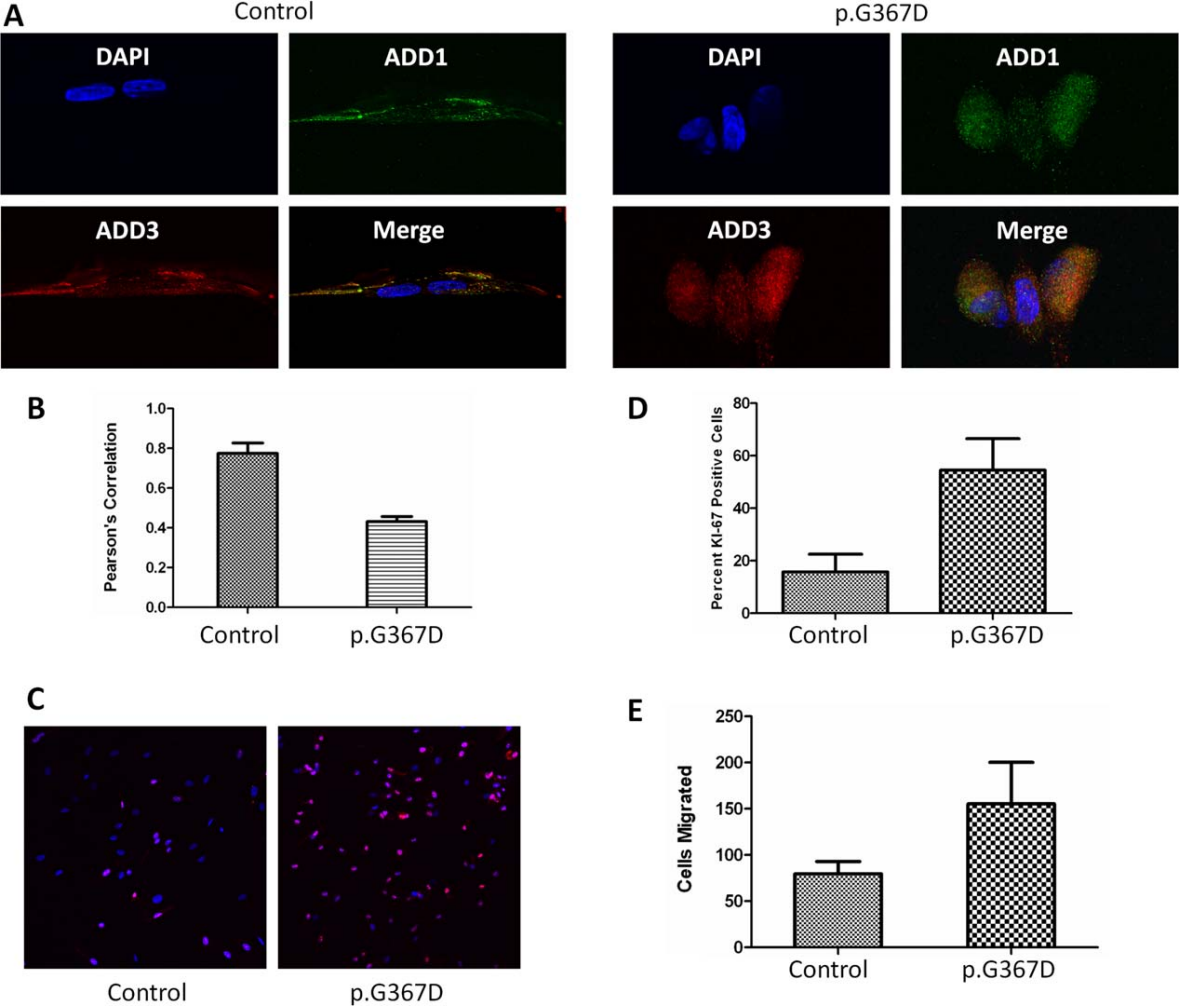
In vitro effects of ADD3 p.G367D. Colocalization of α and γ adducin in wild-type versus ADD3 p.G367D cells. (A) Adducin (α [green] and γ [red]) colocalization in wild-type versus mutant ADD3 fibroblasts. Impaired colocalization is seen in merge as affected fibroblasts (right) show diminished yellow coloration (representative images). (B) Degree of colocalization by Pearson correlation coefficient (mean ± standard deviation [SD]; 3 fields selected at random from 2 independent cultures of each patient or age-matched control; *p* < 0.01 by Student *t* test). Proliferative capacity in oligomerization-defective mutants. (C) Ki-67-positive cells (red) are significantly more abundant in mutant cells (DAPI counterstain). (D) ADD3 p.G367D mutant fibroblasts show increased proliferation by Ki-67 staining as compared to wild type (mean ± SD; 2 coverslips were seeded per patient or age-matched control, and 6 images were analyzed from each coverslip; experiments were performed at least twice; *p* < 0.01 by *t* test). Migration in oligomerization-defective mutants. (E) Mutant cells show increased migration into cell-free region (5 fields selected at random from 2 independent cultures of each patient or age-matched control; mean ± SD, *p* < 0.05 by *t* test). [Color figure can be viewed in the online issue, which is available at http://www.annalsofneurology.org.]

The α adducin oligomerization domain was experimentally determined to include residues 335–436 through studies of truncation mutants.^27^ Within this domain, residues 360–386 are highly conserved between species and adducin isoforms (see Supplementary Fig 1A); this critical region corresponds to residues 363–389 of γ adducin. Furthermore, site-directed mutagenesis of residue 369 was shown to interfere with normal heteromer formation.^27^ Given the potential for the ADD3 pG367D aspartic acid substitution within the oligomerization domain to influence γ adducin interactions with the α subunit, double-labeling experiments were performed. These demonstrated a paucity of colocalization in affected fibroblasts compared to controls (Fig 4A), indicating an impairment of heteromer formation despite adequate protein levels of each isoform. β Adducin expression was not detected in either control or patient fibroblasts, consistent with prior findings (data not shown). Morphologically, mutant fibroblasts demonstrated a lack of neuritelike processes as compared to wild-type fibroblasts (see Fig 4A), consistent with the role of γ adducin in controlling process outgrowth.^30^

### Adducin Function

α Adducin has been shown to influence rates of endocytosis,^31^ cellular migration and proliferation through interactions with protein kinase Cδ,^32^ and mitotic spindles and centrosomes,^11^ respectively, with implications for functional neuronal connectivity. We thus evaluated endocytosis as well as the migrational and proliferative capacity of mutant fibroblasts. Although we found no differences in transferrin endocytosis (Supplementary Fig 3), we found that both proliferation (see Fig 4C, D) and migration (see Fig 4E) were significantly increased in patient cells compared to controls. This may be due to aberrant actin-capping activity. Alternatively, impaired adducin heteromer formation may lead to alterations in nuclear interactions of monomeric adducin. Although additional studies will be required to elucidate the precise mechanisms involved, the gray matter heterotopia seen in Patient II-2 may be related to abnormal migration from the periventricular germinal zone. Furthermore, given the importance of adducin in regulating dendritic spine formation, retraction, and remodeling,^12^ adducin mutations could lead to neuron-specific defects in these processes. These alterations could ultimately lead to impaired functional connectivity, as evidenced by our diffusion tensor imaging findings (see Fig 1F–H), which indicate abnormal fiber tract properties, likely related to disruptions in axonal connectivity, density, or integrity.

### Phenotypic Characterization of Adducin Loss of Function in *Drosophila*

**FIGURE 5:**
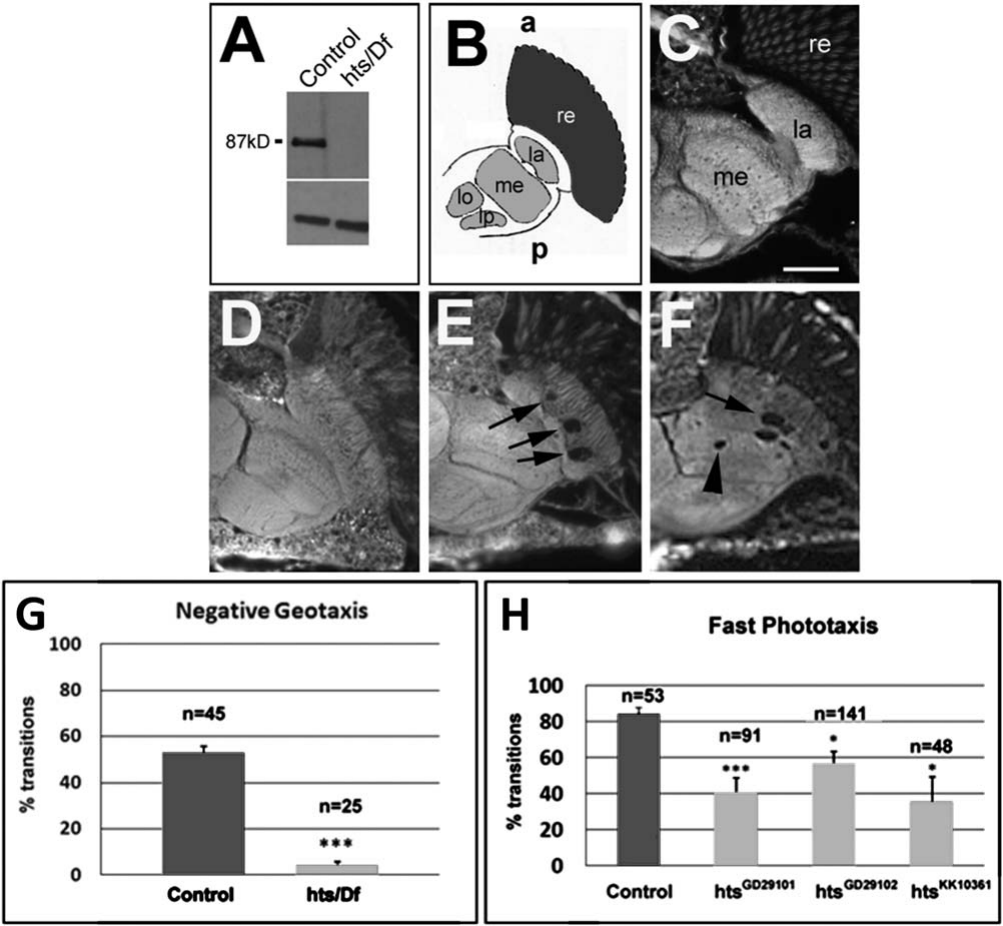
Loss of function of *hts* leads to neuromotor impairment in *Drosophila*. (A) Western blot showing that flies carrying *hts^1103^* over a genomic deletion do not show detectable protein levels. (B) Schematic of a horizontal section of a fly head showing the optic system. The retina (re) is shown in dark gray, whereas the 4 optic neuropils, the lamina (la), medulla (me), lobula (lo), and lobula plate (lp), are shown in light gray. The neuropil consists of axons and dendrites, whereas the cortex region (white) houses neuronal cell bodies. (C) Thirty-day-old wild-type flies have an intact optic system (scale bar = 25μm). (D) At 1 day after eclosing from the pupal case, *hts^1103^*/Df flies do not show any lesions. (E) After 14 days, *hts^1103^*/Df flies have developed vacuoles in the lamina *(arrows)*. (F) Vacuoles extend into the medulla in a 4-week-old fly. (G) Fourteen-day-old *hts^1103^*/Df flies show severe deficits in climbing up a tube (8cm) within 8 seconds. (H) Knocking down *hts* in motor neurons also results in locomotion deficits in the fast phototaxis assay. Standard error of the mean and number of flies tested are indicated; ***p < 0.001, *p < 0.05.

Studies in *Drosophila* have shown an increase in synaptic retractions as well as overgrowth of glutamatergic type Ib boutons with loss of presynaptic hu-li tai shao (*hts*), the *Drosophila* adducin homolog.^33^ We sought to corroborate and extend our proof-of-pathogenesis studies of the p.G367D mutation by evaluating the effects of in vivo loss of function of the *Drosophila* adducin homolog (*hts*^1103^/Df, an *hts* hypomorph). Mutant flies had no detectable hts protein (Fig 5). Lesions of brain lamina and medulla were observed in both *hts^1103^* and *hts* RNAi knockdown. Finally, *hts^1103^* flies displayed impaired locomotion, consistent with a significant role for *adducin*/*hts* in normal neuromotor function.

## Discussion

Prior studies in knockout mice have demonstrated impairment of locomotor function and learning in β adducin knockout mice.^29^ These findings accompanied changes in subunit composition and phosphorylation state, supporting a role for the fine regulation of adducin activity in normal learning and motor function. Additionally, loss of α adducin has been shown to impair feedforward inhibition growth in the developing brain,^34^ with consequences for learning and memory formation, again pointing to a critical role for the adducins in the tightly regulated processes underlying functional central nervous system connectivity. Although γ adducin–null mice have been generated, thus far, no significant red cell abnormalities have been characterized,^35^ but to our knowledge, the brains of these mice have not been studied in detail.

Given the interaction of adducin with spectrin, it is intriguing that mutations in αII spectrin have previously been identified as a cause of epilepsy, intellectual disability, and spastic quadriparesis.^36^ Similar to adducin, KANK1 (also implicated in hereditary CP) plays an important role in actin capping and controls cell migration, cell polarity, and wound healing.^37^,^38^ CP-associated proteins adaptor protein complex-4 (AP-4) and adducin each play an integral role in the egress of vesicle cargoes from the trans-Golgi network.^39^ AP-4 is also known to regulate the recycling of cell-surface α-amino-3-hydroxy-5-methyl-4-isoxazole propionic acid receptors,^40^ and adducin modulates cell surface expression of excitatory amino acid carrier 1,^41^ both key mechanisms controlling glutamatergic neurotransmission in the central nervous system. Taken together, these findings link hereditary forms of CP and implicate abnormalities of components of the dynamic cytoskeleton, process outgrowth, and protein trafficking with neuromotor dysfunction.

In summary, we have identified a homozygous p.G367D mutation in ADD3 in children with spastic diplegic/quadriplegic CP and intellectual disability. Our in vitro studies indicate that the gamma adducin p.G367D mutant impairs heterodimer/heterotetramer formation, disrupting the normally finely modulated regulation of cellular actin polymer growth. The mutation leads to changes in process extension, cell migration, and proliferation, with implications for the pathogenesis of *ADD3*-associated neuromotor impairment. Our in vitro findings are substantiated by our studies in *Drosophila*, which support a critical role for adducin in normal neuromotor function.

Genetic forms of CP are likely to be individually rare, as has been shown for intellectual disability.^42^ This can present a challenge to ongoing efforts to identify relevant disease genes, particularly given findings that suggest that predicted loss of function sequence variants occur at greater than expected frequency in most genomes.^43^ Such studies underscore the importance of following up massively parallel sequencing findings with mechanistic in vitro validation of putative mutations. Evaluating larger cohorts of idiopathic CP patients will shed light on genetic contributions to CP once enough patients are able to be enrolled.

Overall, the identification and characterization of causative genes may identify pathways that are important for normal neuromotor function and help identify new targets for drug screens. Such studies may also shed light on essential biological mechanisms that control neuron–neuron interactions and regulate synaptic plasticity. Finally, increased recognition of a genetic basis for many patients with CP may have far-reaching implications for a condition long presumed to be exclusively caused by prenatal or perinatal injury.
