# Supplementary figures and images for: Mutations in Gamma Adducin are Associated With Inherited Cerebral Palsy
Source: Ann Neurol. 2014 Jan 21;74(6):805–14. doi: 10.1002/ana.23971 (PMC3952628; doi:10.1002/ana.23971)

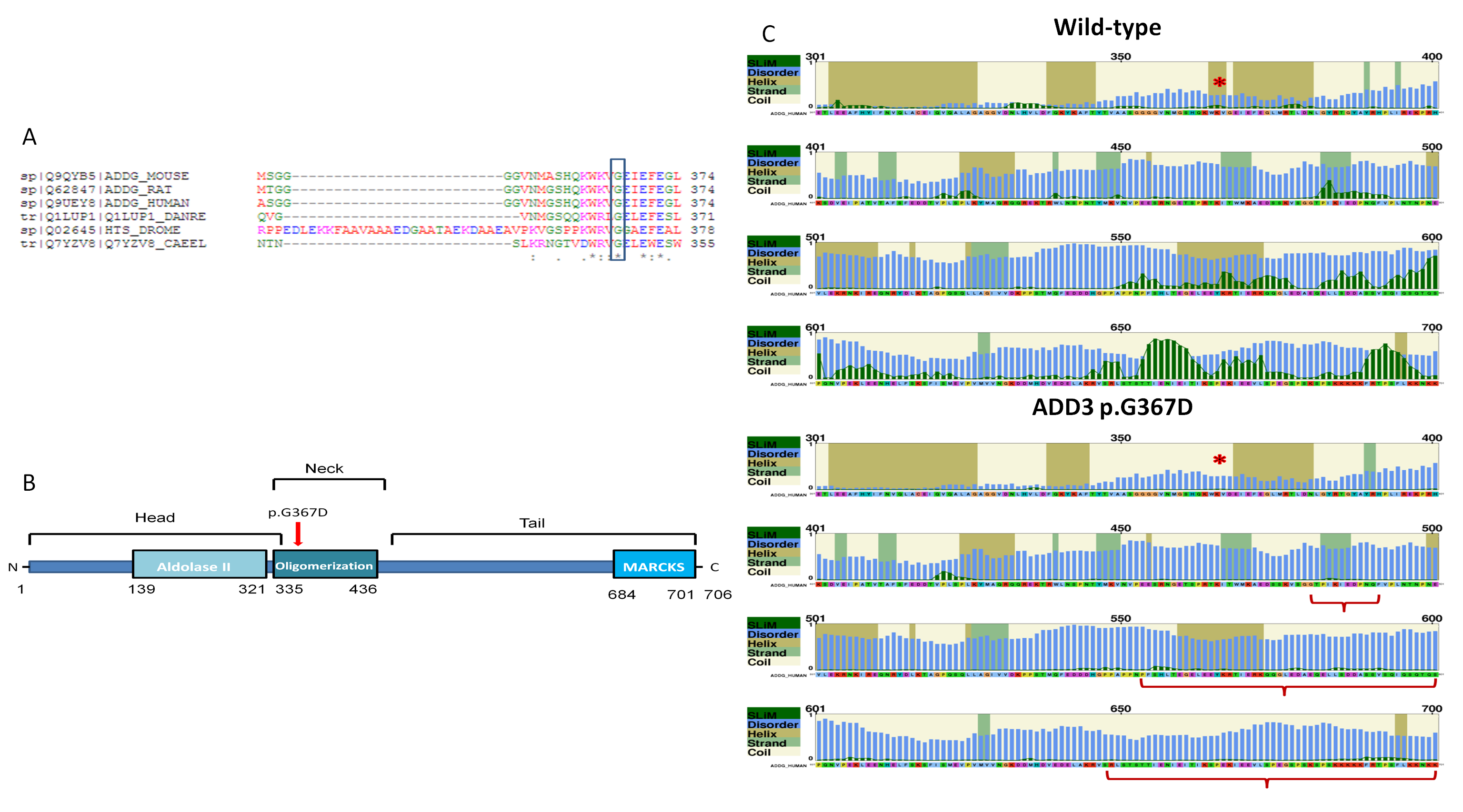

Supplement: Supplementary file 1 — Supporting Information Figure 1 [file ana0074-0805-sd1.tif]

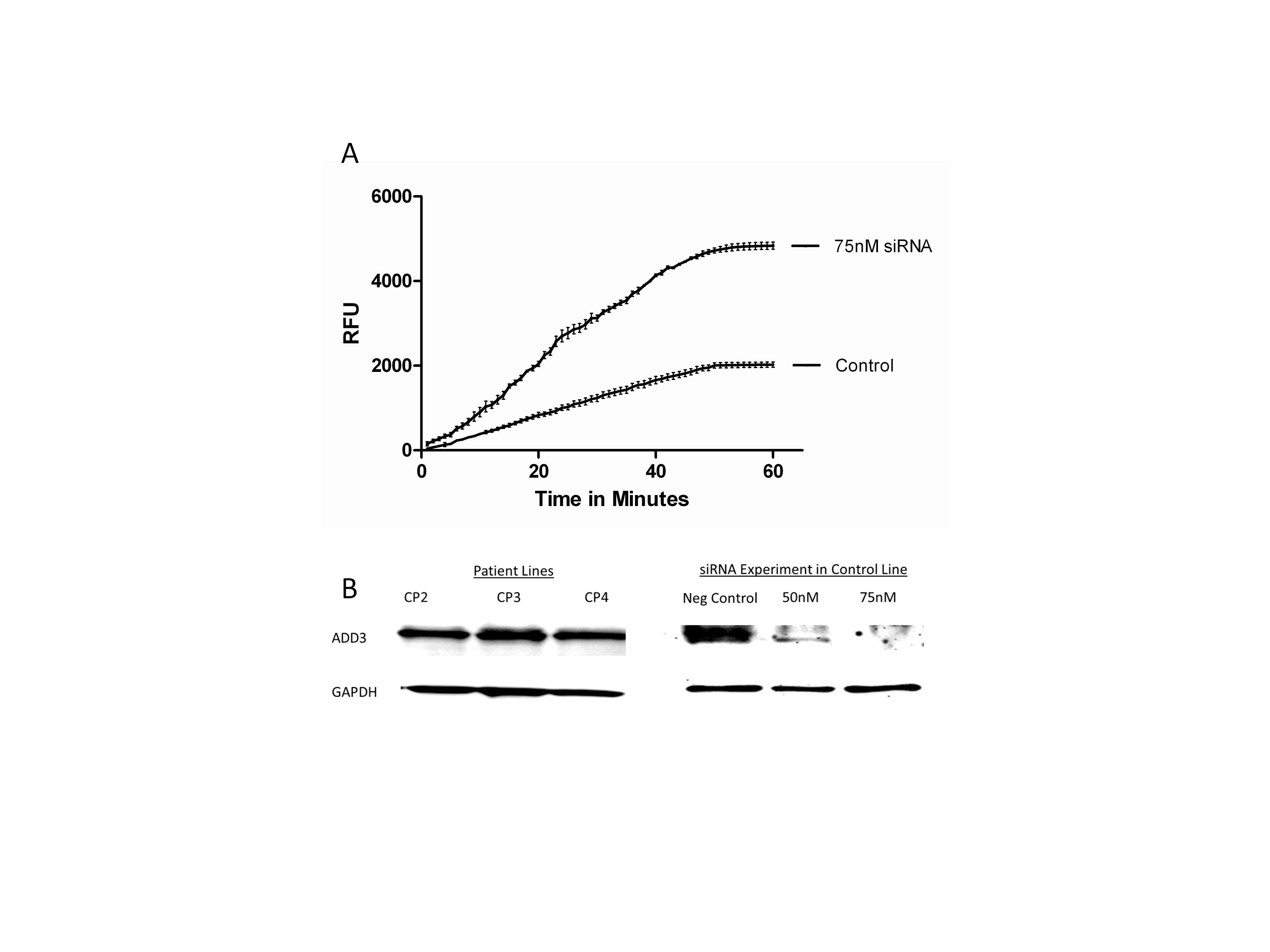

Supplement: Supplementary file 2 — Supporting Information Figure 2 [file ana0074-0805-sd2.tif]

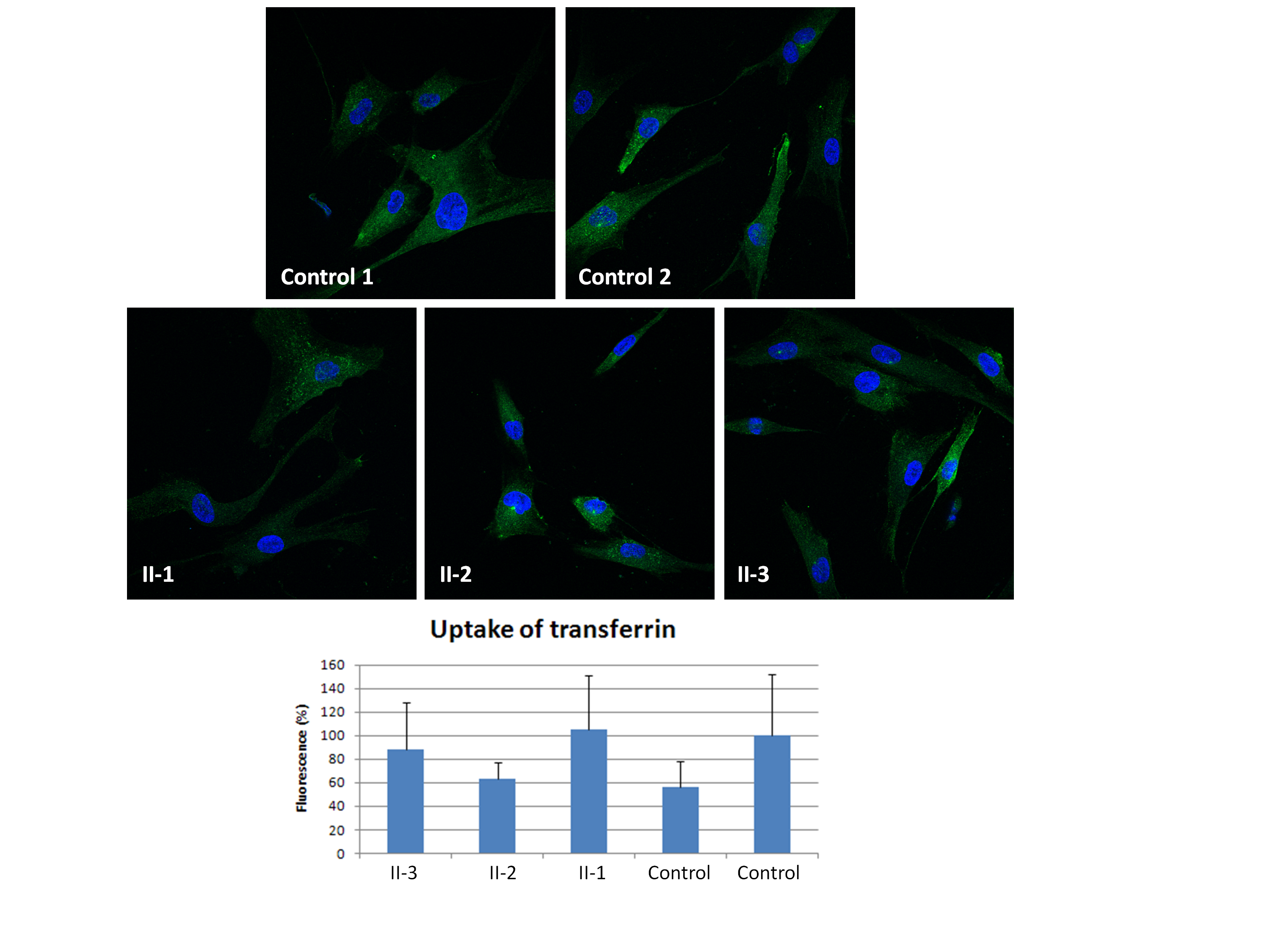

Supplement: Supplementary file 3 — Supporting Information Figure 3 [file ana0074-0805-sd3.tif]
